# High intra- and inter-observer reliability of planning implant size in MRI-based patient-specific instrumentation for total knee arthroplasty

**DOI:** 10.1007/s00167-020-05946-1

**Published:** 2020-03-30

**Authors:** Daphne A. L. Schoenmakers, Dieuwertje M. J. Theeuwen, Martijn G. M. Schotanus, Edwin J. P. Jansen, Emil H. van Haaren, Roel P. M. Hendrickx, Nanne P. Kort

**Affiliations:** 1 Department of Orthopaedic Surgery, Zuyderland Medical Centre, Location Sittard-Geleen, PO Box 5500, 6130 MB Sittard-Geleen, The Netherlands; 2 CortoClinics, Schijndel, The Netherlands

**Keywords:** Total knee arthroplasty, Patient-specific instrumentation, Pre-operative planning, Implant size, Intraclass correlation coefficients

## Abstract

**Purpose:**

Patient-specific instrumentation (PSI) in total knee arthroplasty (TKA) uses individually designed disposable guides to determine intraoperative bone cuts. The manufacturer provides the surgeon with proposed planning which can be modified and should be approved by the surgeon before the guides are produced. This study aims to assess the intra- and inter-observer reliability among preoperative planning by orthopaedic surgeons using PSI. The authors hypothesize a high intra- and inter-observer reliability in planning TKA using PSI.

**Methods:**

Four orthopaedic surgeons modified and approved 40 preoperative MRI-based PSI plannings three times. The surgeons were blinded to their own and each other’s results. Intra- and inter-observer reliability was obtained for planned implant size, resection, and position of the implant.

**Results:**

Intra-observer reliability Intraclass Correlation Coefficients (ICC) were excellent for femoral and tibial implant size with a range of 0.948–0.995 and 0.919–0.988, respectively. Inter-observer reliability for femoral and tibial implant size showed an ICC range of 0.953–0.982 and 0.839-0.951, respectively. Next to implant size, intra- and inter-observer reliability demonstrated good to an excellent agreement (ICC > 0.75) for 7 out of 12 remaining parameters and 6 out of 12 remaining parameters, respectively.

**Conclusion:**

Preoperative planning of TKA implant size using MRI-based PSI showed excellent intra- and inter-observer reliability. Further research on the comparison of predicted implant size preoperatively to intraoperative results is needed.

## Introduction

Patient-specific instrumentation (PSI) in total knee arthroplasty (TKA) uses individually designed, disposable guides, to determine intraoperative bone cuts. Patient-specific preoperative 3D models for the femur and the tibia can be generated either from preoperative magnetic resonance imaging (MRI) or computed tomography scans (CT). A technician is able to make a default plan for the implant size and position using this data. The surgeon can make adjustments to all settings of the femur and tibia component, taking in mind each patient’s anatomical variations. After the case is approved, the manufacturer produces disposable guides for intraoperative use. Previous literature has shown that the plan provided by the technician can differ from the approved plan by the surgeon [4, 10, 12]. Consequently, differences between the suggested and appropriate component size may occur. Therefore, the expertise of the surgeon is essential for evaluating and approving the planning provided by the manufacturer. Nonetheless, none of these studies evaluated the intra- or inter-observer reliability of the planning made by the surgeon.

Multiple other studies have been conducted to assess the radiographic and clinical outcome of conventional TKA compared to PSI [6, 7, 14]. Other studies compared CT- to MRI-based PSI for TKA [1, 13, 16]. However, no literature exists on evaluating the reliability of the planning method itself by comparing repetitive preoperative planning within or between orthopedic surgeons. This comparison is of added value since it demonstrates whether TKA-planning using PSI is itself reliable. Therefore, the present study is designed to assess the intra- and inter-observer reliability among preoperative planning by orthopedic surgeons using PSI. The authors hypothesize that there is a high intra- and inter-observer reliability in planning TKA using PSI.

## Materials and methods

The study group consists of all patients who underwent TKA in 2015 using PSI (Signature™ system, Zimmer-Biomet Inc., Warsaw, IN) based on a preoperative MRI in the Zuyderland Medical Center (Sittard-Geleen, The Netherlands). A total of 309 patients were included. From this cohort, 40 patients were randomly selected and anonymised by Materialise NV (Leuven, Belgium). The preoperative plan, in the default setting as suggested by Materialise NV, had to be evaluated, adjusted where necessary, and approved by the surgeon.

Institutional review board (METC Z, Heerlen, the Netherlands) approval was obtained for this study (trial number 13-N-117).

### Measurements

Four orthopaedic surgeons were each given three folders, within every folder the selected 40 cases in random order. As a result, each surgeon performed standard preoperative planning three times per case within 2 weeks. All surgeons were senior surgeons and had a minimum experience of 3 years with PSI for TKA.

Only the manufacturer had information regarding matching case numbers until the evaluation of all approved plannings. For each case, the following 14 parameters were planned: femoral size, femoral posterior medial resection, femoral mediolateral displacement, femoral distal medial resection, femoral flexion–extension, femoral varus-valgus, femoral rotation from the epicondylar axis, tibial size, tibial anteroposterior displacement, tibial mediolateral displacement, tibial resection from the highest point, tibial posterior slope, tibial varus-valgus, and tibial rotation.

### Outcome measurements

The primary outcome measurements were intra- and inter-observer reliability of planned size component for the femur and tibia. The secondary outcome measurements were intra- and inter-observer reliability of all remaining planned measurements as described above.

### Statistical analysis

All statistical analyses were performed using SPSS software version 25 (SPSS Inc., Chicago, Illinois).

The Intra- and inter-observer reliability of all measurements were determined by Intraclass Correlation Coefficients (ICCs), using an absolute-agreement two-way mixed-effects model for intra-observer reliability, and an absolute-agreement two-way random effects model for inter-observer reliability.

ICC values less than 0.5 are indicative of poor reliability, values between 0.5 and 0.75 indicate moderate reliability, values between 0.75 and 0.9 indicate good reliability, and values greater than 0.90 indicate excellent reliability [9].

## Results

Determination of femoral- and tibial implant size showed excellent agreement with ICCs for intra-observer reliability within a range of 0.948–0.995 and 0.919–0.988, respectively, as well as excellent ICCs for inter-observer reliability within a range of 0.953–0.982 and 0.839–0.951, respectively (Table 1). The maximum size change when an implant size was changed, when compared to other plannings within the same case, was 1 size for the femoral component and 2 sizes for the tibia component. The amount of adjusted implant sizes and differences between the implant sizes within the same case per surgeon are shown in Table 3.

**Table 1:** Intra- and inter-observer Intraclass Correlation Coefficients of surgical parameters in patient-specific TKA

	Intra ICC Surgeon 1	Intra ICC Surgeon 2	Intra ICC Surgeon 3	Intra ICC Surgeon 4	Inter ICC
Femoral distal medial resection	0.902 (0.842–0.943)	1.000	1.000	0.923 (0.874–0.956)	0.919 (0.881–0.950)
Femoral flexion extension	0.595 (0.423–0.742)	0.620 (0.451–0.760)	1.000	0.823 (0.724–0.895)	0.487 (0.369–0.624)
Femoral mediolateral displacement	1.000	0.987 (0.979–0.993)	1.000	1.000	0.997 (0.995–0.998)
Femoral posteromedial resection	1.000	1.000	1.000	1.000	0.998 (0.997–0.999)
Femoral rotation from epicondylar axis	1.000	1.000	1.000	1.000	1.000
Femoral size	0.987 (0.979–0.993)	0.990(0.983–0.994)	0.977(0.959–0.987)	0.966 (0.942–0.981)	0.970 (0.953–0.982)
Femoral varus/valgus	1.000	0.737 (0.604–0.840)	1.000	1.000	0.129 (0.065–0.231)
Tibial anteroposterior displacement	0.737 (0.604–0.839)	0.816 (0.715–0.891)	0.972 (0.954–0.984)	0.942 (0.905–0.967)	0.250 (0.160–0.379)
Tibial mediolateral displacement	0.900 (0.839–0.942)	0.754 (0.626–0.851)	1.000	0.384 (0.186–0.579)	0.177 (0.101–0.294)
Tibial posterior slope	1.000	1.000	1.000	1.000	1.000
Tibial resection from highest point	0.946 (0.911–0.969)	0.939 (0.901–0.965)	0.997 (0.994–0.998)	0.932 (0.889–0.961)	0.743 (0.584–0.851)
Tibial rotation	1.000	1.000	1.000	1.000	1.000
Tibial size	0.973 (0.955–0.985)	0.976 (0.959–0.987)	0.977(0.962–0.987)	0.935(0.867–0.967)	0.910 (0.839–0.951)
Tibial varus/valgus	1.000	0.560 (0.383–0.715)	1.000	1.000	0.081 (0.032–0.164)

Intraclass Correlation Coefficients (95% confidence interval)

*Intra ICC,* intra-observer intraclass correlation coefficients; *Inter ICC,* inter-observer intraclass correlation coefficients

**Fig. 1:**
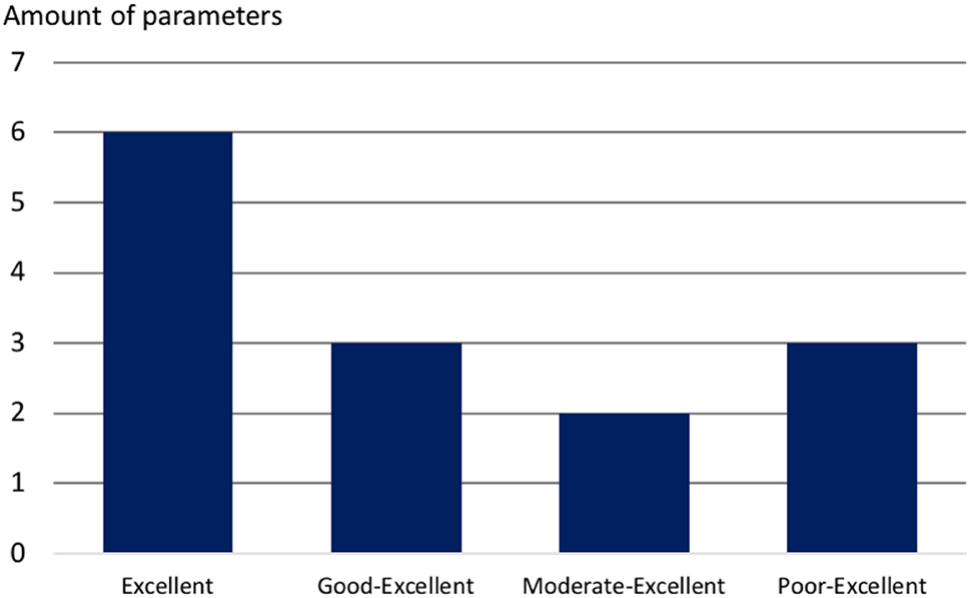
Number of parameters in each intra-observer reliability range

**Fig. 2:**
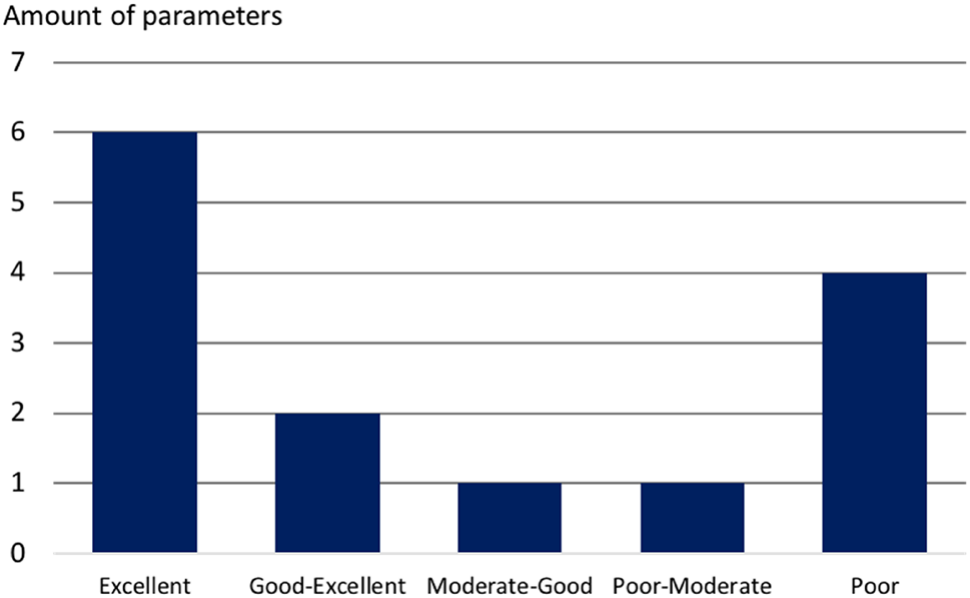
Number of parameters in each inter-observer reliability range

Furthermore, intra- and interobserver reliability demonstrated excellent to good agreement (ICC > 0.75) for 7 out of 12 remaining parameters, and 6 out of 12 remaining parameters, respectively. A different agreement per surgeon, with an intra-observer reliability ranged from moderate to excellent (ICC range > 0.5–> 0.9) was found in 2 out of 12 parameters, as well as in the range from poor to excellent (ICC range < 0.5–> 0.9) (Figs. 1 and 2).

For 3 parameters (femoral rotation from the epicondylar axis, posterior tibial slope, tibial rotation) no changes to the proposed planning were made by any of the surgeons, resulting in an intra- and inter-observer reliability ICC of 1.00.

Table 2 shows an overview per case of changes from proposed planning and alterations within adjusted plannings. All modifications per surgeon are listed in Table 3.

**Table 2:** Amount of adjusted cases combined for all surgeons

	Total number of cases adjusted from default plan *n* (%)	Adjusted plans within same case identical *n*
Femoral distal medial resection	16 (10%)	1
Femoral flexion extension	60 (37.5%)	22
Femoral mediolateral displacement	1 (0.6%)	N/A
Femoral posteromedial resection	1 (0.6%)	1
Femoral rotation from epicondylar axis	0 (0%)	N/A
Femoral size	52 (32.5%)	13
Femoral varus/valgus	4 (2.5%)	2
Tibial anteroposterior displacement	73 (45.6%)	2
Tibial mediolateral displacement	77 (48.1%)	2
Tibial posterior slope	0 (0%)	N/A
Tibial resection from highest point	96 (60%)	54
Tibial rotation	0 (0%)	N/A
Tibial size	63 (39.4%)	28
Tibial varus/valgus	19 (11.9%)	6

*n,* Number of adjusted cases, or number of identical adjusted plans within same case; *N/A,* not applicable

**Table 3:** Amount of adjusted cases and differences between adjusted plans within the same case per surgeon

	Surgeon 1		Surgeon 2		Surgeon 3		Surgeon 4	
	*n* (%)	Difference	*n* (%)	Difference	*n* (%)	Difference	*n* (%)	Difference
Femoral distal medial resection	7 (17.5%)	1 mm: 4 1.5 mm: 1 2 mm: 2	0 (0%)	N/A	0 (0%)	N/A	9 (22.5%)	0 mm: 1 1 mm: 3 1.5 mm: 2 2 mm: 3
Femoral flexion extension	18 (45%)	0 dg: 2 1 dg: 10 2 dg: 4 2.2 dg: 1 2.5 dg: 1	38 (95%)	0 dg: 20 0.5 dg: 15 1 dg: 3	0 (0%)	N/A	4 (10%)	0.7 dg: 1 0.8 dg: 1 1 dg: 1 1.5 dg:1
Femoral mediolateral displacement	0 (0%)	N/A	1 (2.5%)	2.3 mm: 1	0 (0%)	N/A	0	N/A
Femoral posteromedial resection	0 (0%)	N/A	1 (2.5%)	0 mm: 1	0 (0%)	N/A	0	N/A
Femoral rotation from epicondylar axis	0 (0%)	N/A	0 (0%)	N/A	0 (0%)	N/A	0	N/A
Femoral size	8 (20%)	0 size: 2 1 size: 6	5 (12.5%)	1 size: 5	14 (35%)	0 size: 3 1 size: 11	25 (62.5%)	0 size: 8 1 size: 17
Femoral varus/valgus	0 (0%)	N/A	4 (10%)	0 dg: 2 0.5 dg: 2	0 (0%)	N/A	0 (0%)	N/A
Tibial anteroposterior displacement	35 (87.5%)	0 mm: 1 0.1–0.5 mm: 14 0.6–1.0 mm: 16 1.1–1.5 mm: 3 3.2 mm: 1	33 (82.5%)	0 mm: 1 0.1–0.5 mm: 14 0.6–1.0 mm: 15 1.1–1.5 mm: 3	1 (2.5%)	1.7 mm: 1	4 (10%)	0.7 mm: 2 0.8 mm: 1 1.5 mm: 1
Tibial mediolateral displacement	37 (92.5%)	0 mm: 2 0.1–0.5 mm: 8 0.6–1.0 mm: 14 1.1–1.5 mm: 4 1.6–2.0 mm: 7 2.1 mm: 1 2.6 mm: 1	36 (90%)	0.1–0.5 mm: 15 0.6–1.0 mm: 13 1.1–1.5 mm: 6 1.6–2.0 mm: 2	0 (0%)	N/A	4 (10%)	2.5 mm: 1 2.6 mm: 1 6.2 mm: 1 7.3 mm: 1
Tibial posterior slope	0 (0%)	N/A	0 (0%)	N/A	0 (0%)	N/A	0 (0%)	N/A
Tibial resection from highest point	36 (90%)	0 mm: 27 2 mm: 9	40 (100%)	0 mm: 19 0.5 mm: 6 1 mm: 12 1.5 mm: 1 2 mm: 2	1 (2.5%)	1 mm: 1	19 (47.5%)	0 mm: 8 1 mm: 3 2 mm: 8
Tibial rotation	0 (0%)	N/A	0 (0%)	N/A	0 (0%)	N/A	0 (0%)	N/A
Tibial size	13 (32.5%)	0 size: 6 1 size 7	10 (25%)	0 size: 3 1 size: 7	7 (17.5%)	1 size: 7	33 (82.5%)	0 size: 19 1 size: 12 2 sizes: 2
Tibial varus/valgus	1 (2.5%)	1 dg: 1	17 (42.5%)	0 dg: 6 0.5 dg: 11	0 (0%)	N/A	1 (2.5%)	2 dg: 1

*n,* Number of adjusted cases; *N/A,* not applicable; *dg,* degree(s)

## Discussion

This study shows that planning of TKA using PSI by different surgeons results in an excellent agreement for implant sizes between surgeons as well as in repeated planning by the same surgeon. Next to implant size, intra- and interobserver reliability demonstrated good to excellent agreement (ICC > 0.75) for 7 out of 12 remaining settings and 6 out of 12 parameters, respectively. Hence, it may be stated that PSI is a reliable method for planning of a TKA.

Previous studies have shown that PSI planning accurately predicts the implant size used intraoperatively [5, 8, 12]. The current study shows that planning of the implant size, within and between orthopaedic surgeons, is reliable. The maximum size difference was 1 implant size for the femur, and 2 implant sizes for the tibia, compared to the other plannings of the same patient.

Changes to the default plan can result in different implant sizes. Overall, more changes were made to the tibia than the femur. This may explain the greater difference in default and approved implant size for the femur and tibia: 1 size versus 2 sizes, respectively. TKA surgery can be planned more effectively by understanding the size change frequency of implants, in combination with intraoperative concordance to the preoperative plan. Consequently, when implant sizes can be accurately predicted, and planning implant sizes itself is reliable, the operating team will be able to minimize intraoperative implant size errors in advance. This may lead to improved operating room efficiency due to a decreased number of required operative trays (eq. reliable patient-specific trays), less inventory planning, and possibly lowering hospital costs per TKA procedure. This may be of interest for future research.

Previous studies have emphasized that changes in the initial technician’s plan were necessary to get an accurate preoperative planning of the implant sizes. Intraoperative alterations in implant size were significantly lower for the plans approved by the surgeon compared to the default plans provided by the technician [4, 10, 12]. Based on this previous literature, the expertise of the surgeon is thus essential for evaluating and approving the default planning provided by the manufacturer.

Intra- and inter-observer reliability ICC were 1.00 for femoral rotation from the epicondylar axis, posterior tibial slope, and tibial rotation because none of the surgeons modified these parameters. Due to general consensus on these parameters, less variation will occur, with a higher agreement as result. When there is less consensus on a certain parameter, more changes are made which will result in a lower agreement. Surgeon 3 made the fewest alterations to the proposed plannings, resulting in an excellent agreement (ICC > 0.90) for intra-observer reliability for all settings. Thus, high ICC can be caused by good agreement between adjusted plannings, or due to no alterations made to the proposed plan. Additionally, the adjustment of one parameter can derive alterations of other parameters. For example, an increase of resection might result in the need for a smaller implant size and adjustments in placement of the newly chosen implant size. Awareness of this effect is essential when interpreting the results of this article. This ‘snowball effect’, as well as less consensus on certain parameters with more changes to the default planning and therefore more differences within and between surgeons, may explain why parameter such as femoral flexion/extension and tibial displacement showed less agreement.

Mechanical alignment technique is considered well performed when the overall limb alignment is within 3° of neutral. Varus- and valgus angles for both femur and tibia showed modifications with a maximum of 0.5° and 2° respectively. Given that the maximum difference of varus/valgus angles is 2° within the same case, it is supposed that these changes are of no clinical importance. Moreover, adjustments to varus/valgus alignment are known to be dependent on the surgeon’s philosophy for an anatomical-, (adjusted) mechanical-, or (restricted) kinematic alignment technique [2, 11]. A patient’s specifications, such as findings from a physical examination (for example preoperative leg axis, body mass index, and laxity) and previous medical history, can be determinative in the decision for a certain alignment.

Patient-specific characteristics uncontrollable by planning software, namely, ligamentous balancing and lower limb alignment, can require intraoperative changes. Therefore, correct matching of the pre-operative plan and intraoperative observations is a crucial factor in PSI-assisted TKA. In case of a mismatch, it is the surgeon’s responsibility to consider a switch to conventional instrumentation. In previous literature intra-operative modifications were made to the pre-operative plan in 23% up to 36% of MRI-based and CT-based PSI-assisted TKA, respectively. Most of these changes occurred due to a poor match between the pre-operative plan and intra-operative observations for the tibial component [3, 4, 15].

Furthermore, each surgeon has a ‘personal touch’ not only in planning, but also intraoperatively with his or her own preferences of additional releases, the decision whether or not recuts are needed, or the consideration to select a thicker insert in patients with a high BMI. Nonetheless, excellent agreement for implant sizes between surgeons and within surgeons was found in this study. Also, the agreement of implant size did not differ between the surgeons who made multiple changes to the proposed plans compared to the surgeon who made very little changes to the proposed plan.

This study has some limitations. Firstly, no power analysis for the number of surgeons, the number of patient cases, and repetitive measurements have been conducted. However, Koo et al. suggested as a rule of thumb that researchers should obtain at least 30 heterogeneous samples and involve at least 3 observers whenever possible when conducting a reliability study [9]. Therefore, in the present study 40 patients were included and planned by 4 different orthopedic surgeons. Secondly, only one type of PSI was evaluated in this study. Other PSI planning systems may perform differently. Thus, these results may not be representative of all PSI technologies available. No comparison to the intraoperative results was made during this study; this study focused on the agreement of repeated planning by different surgeons—the correlation between the planned and placed implant has already been addressed in previous studies.

This study represents the first assessment of intra- and inter-observer reliability in PSI TKA. The study showed an excellent intra- and inter-observer reliability, among which implant sizes. This may contribute to more optimal and potentially effective preoperative planning of TKA surgery in the future. Therefore, this topic can be of interest for further research. Future research, with larger dataset measurements and different types of both MRI- and CT-based PSI, is necessary to further evaluate these results.

## Conclusions

Preoperative planning of TKA implant size using MRI-based PSI showed excellent intra- and inter-observer reliability. Future research on the comparison of predicted implant size preoperatively to intraoperative results is needed.
